# Rapid in vitro regeneration method for *Moringa oleifera* and performance evaluation of field grown nutritionally enriched tissue cultured plants

**DOI:** 10.1007/s13205-012-0045-9

**Published:** 2012-02-04

**Authors:** R. K. Saini, N. P. Shetty, P. Giridhar, G. A. Ravishankar

**Affiliations:** Plant Cell Biotechnology Department, Council of Scientific and Industrial Research, Central Food Technological Research Institute, CFTRI, Mysore, 570 020 India

**Keywords:** Carotenoids, Drumstick, Horseradish tree, Micropropagation, Tocopherol

## Abstract

The present investigations were attempted to develop the rapid in vitro micropropagation protocol of *Moringa oleifera* (Variety-PKM-1) from nodal sections of young, aseptically grown seedlings. Benzyladenine (BA) at 4.44 μM was found to be optimal in producing on maximum an average of 9.0 ± 1.0 axillary shoots per explant after 15 days of inoculation. A high multiplication rate was established through routine sub culturing of nodal sections explanted from in vitro shoot cultures. In vitro rooting of individual shoot culture was maximum (100%) on medium containing indole-3-acetic acid (IAA) at 2.85 μM along with indole-3-butyric acid (IBA) at 4.92 μM. Eighty percent of the rooted plants survived after being transplanted in the soil, provided that the potted plantlets were covered with clear polythene bags and kept in a shaded greenhouse for 15 days before exposure to ambient conditions. Fresh leaves of field grown tissue culture plants were analyzed for lutein, β-carotene, α-tocopherol, total carotenoids and chlorophyll content. Tissue culture-derived plants were found nutritionally superior over control plants to contain 13.2 and 14.7% higher amount α-tocopherol and total carotenoids, respectively. The result of present study will be useful for rapid clonal propagation of *M. oleifera* and production of nutritionally superior plant.

## Introduction

*Moringa oleifera* Lam. commonly known as the drumstick or ben oil tree is a widely cultivated species of monogeneric family Moringaceae and native to the sub-Himalayan tracts of Northwestern India. It is a fast-growing tropical perennial soft-wooded tree with a long history of traditional medicine and culinary uses. It is widely cultivated in India, the Philippines, Sudan, South Africa, tropical Asia, Latin America, the Caribbean, and in the Pacific islands (Verdcourt 1985; Palada 1996). Other species of genus *Moringa* are: *M*. *stenopetala* is an important crop in Kenya and Ethiopia (Verdcourt 1985). Similarly, *M. peregrina* was known to the ancient Egyptians who utilized its seed oil. All of the other 10 species of this genus are reported to be having pharmacologic properties (Morton 1991; Olson 2001); however, some are in danger of extinction, specially *M.hildebrandtii* is now extinct in the wild (Olson and Razafimandimbison 2000).

*Moringa oleifera* is a promising food source especially it’s leaves which are rich in nutrients and minerals and the tree has maximum leaves at the end of the dry season when other foods are typically scarce (Fuglie 1999). There are tremendous potential opportunities with *M. oleifera* for sustainable agriculture, and the development of cash crops in semiarid regions. The few reports on the tissue culture of *M. oleifera* described clonal propagation through the use of nodal explants taken from non-aseptic sources, either from young seedlings or mature plants (Stephenson and Fahey 2004; Islam et al. 2005; Marfori 2010). The preservation of the *Moringa* species is thus of great concern from biodiversity, ethnobotanical, dietary and pharmacological perspectives.

Our aim of the present study was to develop rapid in vitro regeneration from nodal section of aseptically grown seedlings of *M. oleifera* and evaluation of performance of tissue-cultured plants in field condition.

## Materials and methods

### Plant material

Healthy uniform seeds of *M. oleifera* (Variety-PKM1) were obtained from University of Agricultural Sciences, Bangalore, India. Seeds were surface sterilized inside the laminar flow hood by immersion in 0.1% mercuric chloride (w/v) for 2 min and 20% sodium hypochlorite (v/v) for 10 min, followed by rinsing three times in sterile distilled water. Seed coats were removed aseptically and seeds were again surface sterilized by immersion in 20% sodium hypochlorite (v/v) for 5 min, followed by rinsing three times in sterile distilled water. Seeds were planted aseptically in MS basal medium (Murashige and Skoog 1962) containing 30 g L^−1^ sucrose and solidified with 5 g L^−1^ agar (Himedia). The pH was adjusted to 5.8, after which the medium was dispensed at 40 mL each in culture bottles and sterilized by autoclaving at 121 °C for 20 min. Seed cultures were maintained in the dark at 27 ± 1 °C for 15 days. Upon germination, seedlings were transferred under continuous light at 2,000-Lux intensity produced from cool white fluorescent tubes.

### Induction of multiple shoots

Germinated seedlings consisting of 3–4 nodes (3–4 weeks after inoculation) were used in the experiment. Nodal explants were prepared and transferred to a multiple shoot induction medium (MSI) consisting of MS salts and Triacontanol (TRIA) at 0–11.39 nano molar (nM), benzyl adenine (BA) at 0–8.88 μM and naphthalene acetic acid (NAA) at 0–5.37 μM to determine their effect on multiple axillary shoot formation. All growth regulators used in the study were obtained from Sigma Chemicals Co., St. Louis, MO, USA. Percentage of response, number of shoots per explants and shoot length were recorded 15 days after transfer to MSI. Micro shoots obtained were repeatedly subcultured in MS basal medium supplemented with 4.44 μM BA.

### Rooting of shoots

Nodal sections with induced axillary shoots were transferred to a root induction medium (RIM) consisting of MS salts, indole-3-acetic acid (IAA) at 0–5.71 μM with and without indole-3-butyric acid (IBA) at 0–4.92 μM. Percentage of response, number of roots per shoot and root length were recorded 7 days after transfer to RIM. Rooted plantlets were transferred to the soil for hardening.

### Hardening of the rooted plantlet

Hardening of the rooted plantlet was done in plastic bags containing autoclaved mixture of soil, sand and vermicompost (3:1:1 v/v). Plants were watered, then covered with transparent polythene bags, and kept under partial sunlight inside a greenhouse at ambient temperature (~26–28 °C). After 15 days, the polythene bags were removed and the survived plants were maintained inside the greenhouse for another 15 days. These hardened plantlets were transferred into the field. Performance of these plants was evaluated by measuring the nutrient composition (lutein, β-carotene, α-tocopherol and total carotenoids) and chlorophyll content compared with control plants. For field performance, 1-year old in vitro plant that repeatedly subcultured and in vitro rooted followed by hardening in green house was used upon its field transfer. Control plants were propagated from seeds and grown in field. These 1-year-old control plants were selected for comparison with tissue culture-derived plants, to evaluate the superiority of tissue culture-derived plants over control plants for nutrient composition.

### Extraction of carotenoids and *α*-tocopherol

One gram of fresh leaves sample from tissue cultured and control plants was homogenized in chilled acetone and the extraction was repeated until the samples became colorless (total volume 15 ml).The extracts were then centrifuged at 8,000*g* and filtered through a 0.45-um membrane (Nupore, India). A volume of 20 μL extract was injected into the HPLC system without saponification. The content of carotenoids (lutein and β-carotene) and *α*-tocopherol is expressed as mg/100 g fresh weight.

### HPLC analysis of carotenoids and *α*-tocopherol

Carotenoids and tocopherol were analysed according to Darnoko et al. (2000). The HPLC system consisted of a Shimadzu chromatograph (LC 10-AS HPLC), equipped with dual pump and UV detector (SPD 10A). The column was a YMC C_30_ Carotenoid column 250 × 4.6 mm, 5 mm (YMC, Wilmington, NC). The mobile phase for this column was 81:15:4 methanol: methyl tertiary butyl ether (MTBE): Water (solvent A) and 91:9 MTBE: methanol (solvent B). The gradient elution was 0–50% B in 45 min followed by 0% B in the next 5 min at a flow rate of 1 mL/min. Carotenoids (lutein and β-carotene) were monitored at 450 nm and *α*-tocopherol at 295 nm.

### Chlorophylls and total carotenoids

Chlorophylls and carotenoids were measured and characterized by UV–VIS spectroscopy according to Lichtenthaler and Wellburn (1985). In an acetone extract, chlorophyll *a* (Chl *a*) showed the maximum absorbance at 661.6 nm, chlorophyll *b* (Chl *b*) at 644.8 nm and total carotenoids at 470 nm and the concentrations of Chl *a*, Chl *b* and the sum of carotenoids (cx + c) was calculated using the following equations for acetone extraction, where the pigment concentrations are given in μg/mL extract solution.

### Design and data analysis

Experiments were laid out in a completely randomized block design (CRBD). Each treatment was replicated 3 times. Values from triplicate determinations of each sample were averaged and represent as mean ± standard deviation (SD). The data were analyzed statistically by analysis of variance (ANOVA), and the difference between the mean of sample was analyzed by the least significant difference (LSD) test at a probability level of 0.05.

## Results and discussion

### Influence of growth regulators on shoot multiplication and root formation

**Fig. 1:**
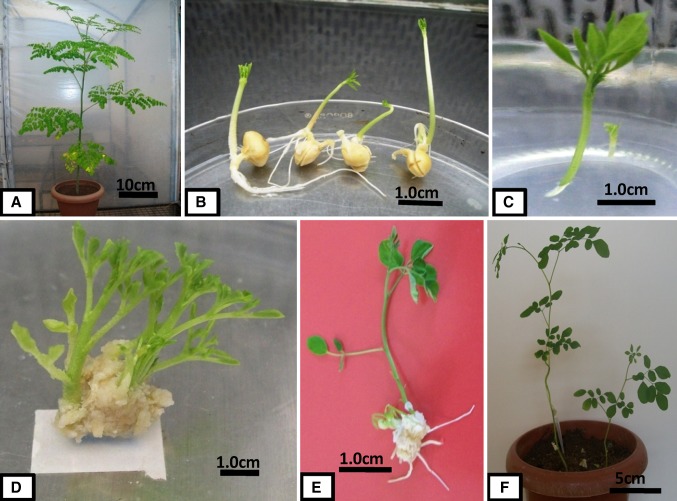
In vitro regeneration of *Moringa oleifera*. **a** Plant grown in green house, **b** aseptically grown seedlings obtained on MS basal medium, **c** elongation of shoot tips on MS basal medium, and **d** multiple shoots obtained from nodal explants on MS medium supplemented with 4.44 μM BA. **e** Rooted plant obtained on MS medium supplemented with 2.85 μM IAA plus IBA at 4.92 μM. **f** Hardened plants in green house

The number of axillary shoots per nodal explant induced by BA, Triacontanol and NAA at various concentrations after 15 days is shown in Table 1. Application of 4.44 μM BA resulted in the highest number of induced axillary shoots (9.0 ± 1.0) per nodal explants (Fig. 1). At lower concentrations, triacontanol (5.7 nM) and NAA (2.69 μM) were less effective than BA in inducing axillary shoots per explant. Triacontanol was most effective at 11.39 nM, inducing an average of 6.0 ± 1.0 axillary shoots per explant.

**Table 1:** Effect of Triacontanol (TRIA), NAA and BA on shoot proliferation from nodal explants of *M. oleifera*L

BA (μM)	TRIA (nM)	NAA (μM)	Percentage of response	No. of shoots per shoot*	Shoot length (cm)*
0.00	0.00	0.00	50^d^	3.0 ± 1.00^cd^	2.16 ± 0.76^cd^
0.00	5.70	0.00	62^c^	3.3 ± 0.57^c^	2.1 ± 0.35^cd^
0.00	11.39	0.00	70^bc^	6.0 ± 1.0^b^	3.16 ± 0.76^c^
0.00	22.79	0.00	65^c^	5 ± 1.0^bc^	2.5 ± 0.5^cd^
4.44	0.00	0.00	94^a^	9.0 ± 1.0a	5.75 ± 0.35^a^
4.44	11.39	0.00	75^bc^	6.3 ± 0.57^b^	3.26 ± 0.76^c^
8.88	0.00	0.00	90^a^	8.3 ± 0.57^a^	4.5 ± 0.81^b^
8.88	0.00	2.69	63^c^	2.6 ± 0.5^cd^	2.1 ± 0.2^d^
8.88	0.00	5.37	60^cd^	2.3 ± 0.58^cd^	2.0 ± 0.3^d^

* Value represents the mean ± standard deviation of three replicates. In a column, different letters indicate statistically significant differences between the mean (*P* < 0.05)

Nodal sections with axillary shoots were transferred to root induction medium with IAA or IBA. Application of 2.85 μM IAA along with IBA at 4.92 μM resulted in the highest number of induced roots (15 ± 1.3 per shoot) after 7 days (Table 2). IAA and IBA alone found less effective at same concentration with 6.3 ± 0.6 and 9.6 ± 0.57 roots per shoot, respectively. IAA was found less effective than IBA for rooting. In all media with plant growth regulator used in the present study, a single axillary shoot was invariably produced with moderate friable callusing at the cut tissue surface in direct contact with the medium.

**Table 2:** In vitro rooting in *M. oleifera* microshoots, on a medium containing different concentrations of IAA and IBA

IAA (μM)	IBA (μM)	% Rooting	No. of roots per shoot*	Root length (cm)*
0.00	0.00	35^cd^	5.6 ± 1.1^cd^	3.8 ± 0.4^c^
0.00	4.92	82^ab^	9.6 ± 0.57^bc^	6.5 ± 0.6^ab^
0.00	9.84	75^b^	8.6 ± 0.57^c^	2.8 ± 0.7^cd^
2.85	4.92	100^a^	15 ± 1.3^a^	8 ± 0.8^a^
2.85	0.00	40^c^	6.3 ± 0.6^cd^	4.2 ± 0.3^c^
5.71	0.00	35^cd^	5.6 ± 0.7^cd^	3.5 ± 0.4^c^

* Value represents the mean ± standard deviation of three replicates. In a column, different letters indicate statistically significant differences between the mean (*P* < 0.05)

Induction of multiple shooting and rooting in *M. oleifera* has been previously characterized with different growth regulators. Stephenson and Fahey (2004) obtained 4.7 shoots per cultured seed in medium containing 1 mgL^−1^ BA with 1 mgL^−1 ^GA_3_, similarly rooting was obtained in ½ MS containing 0.5 mgL^−1^ NAA. According to Islam et al. (2005) 4.44–6.66 μM BA was found to be best for shooting response, whereas rooting was efficient on MS basal medium. The efficiency of BA for organogenesis was further supported by similar reports in other plants (Khan et al. 2011; Yapo et al. 2011). In order to obtain in vitro organogenesis in *Moringa*, nodal explants (Stephenson and Fahey 2004) and stem explants from in vitro seedlings and field-grown plants were, respectively, used. Our results of shoot induction were similar to Stephenson and Fahey (2004) and Islam et al. (2005), but we got more number of shoots per explant compare to earlier studies in *M. oleifera*. In root induction study, we used combination of IBA and IAA, and it was found better than individual performance of these growth regulators. Earlier, no reports are available on the use of such combination of the plant growth regulators to induce the roots in *M. oleifera.*

### Nutrient composition of tissue culture-derived plants

**Fig. 2:**
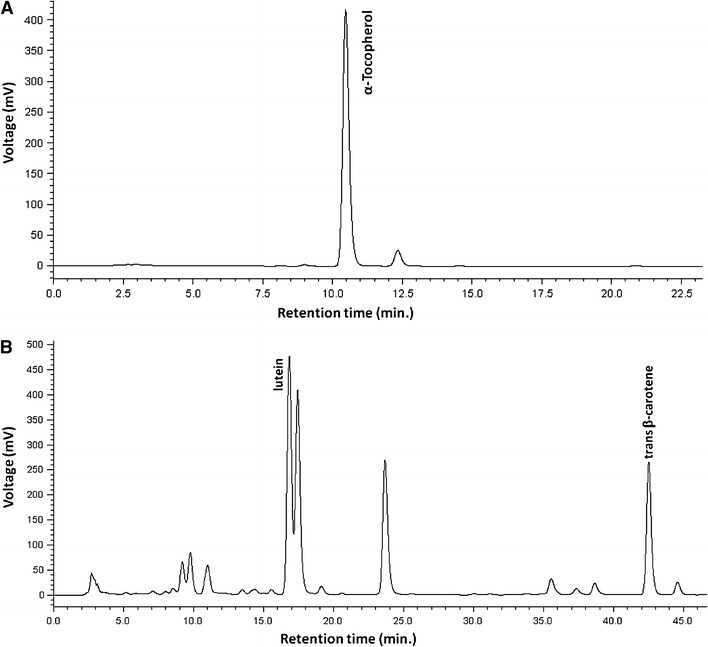
HPLC chromatograms obtained for tocopherol and carotenoid in YMC C_30_ Carotenoid column 250 × 4.6 mm, 5 mm (YMC, Wilmington, NC). **a** Standard α-tocopherol, **b** carotenoids of *Moringa oleifera*

Fresh leaves of tissue cultured and control plant (1-year old) were analyzed by HPLC for lutein (RT-17.0 min), *β*-carotene (RT-42.5 min) and α-tocopherol (RT-10.8 min), the gradient elution system applied in this study provided good resolution, precision, and repeatability (Fig. 2). Study revealed (*P* < 0.05) significantly 5.5–14.7% higher amount of nutrients in tissue culture-derived plants compare to control. Among control and tissue culture-derived plants screened, tissue-cultured plant found to contain (per 100 g FW) 37.96 ± 0.74 mg lutein, 18.86 ± 1.42 mg β-carotene, 41.06 ± 0.70 mg α-tocopherol, 87.20 ± 1.61 mg chlorophyll *a*, 68.73 ± 1.86 mg chlorophyll *b* and 90.78 ± 0.94 mg of total carotenoids. Whereas, conventionally grown (control) plant found to contain (per 100 g FW) 35.99 ± 0.90 mg lutein, 17.78 ± 0.63 mg β-carotene, 36.27 ± 0.91 mg α-tocopherol, 76.01 ± 1.63 mg chlorophyll *a*, 63.86 ± 1.50 mg chlorophyll *b* and 79.17 ± 1.54 mg of total carotenoids (Table 3).

**Table 3:** Nutrient composition of tissue culture derived and control plants (mg/100 g FW) analysed by HPLC and spectrophotometry

Compound analysed	Control plant	Tissue cultured plant	% increase over control
Lutein	35.99 ± 0.90^b^	37.96 ± 0.74^a^	5.5
β-carotene	17.78 ± 0.63^b^	18.86 ± 1.42^a^	6.1
α-Tocopherol	36.27 ± 0.91^ab^	41.06 ± 0.70^a^	13.2
Chlorophyll *a*	76.01 ± 1.63^ab^	87.20 ± 1.61^a^	14.7
Chlorophyll *b*	63.86 ± 1.5^ab^	68.73 ± 1.86^a^	7.6
Total carotenoids	79.17 ± 1.54^ab^	90.78 ± 0.94^a^	14.7

Value represents the mean ± standard deviation of three replicates. In a row, different letters indicate statistically significant differences between the mean (*P* < 0.05)

In the present study, tissue culture-derived field grown plants were found to be superior over control plants for their nutrient and chlorophyll content, wherein 13.2 and 14.7% higher amount of α-tocopherol and total carotenoids were found, respectively. These observations are in agreement with the similar results which indicate that micro propagated plants are rich in nutrients compared to conventionally grown plants (Debnath 2009; Faisal and Anis 2006). In strawberry (*Fragaria ananassa*), total anthocyanin contents and antioxidant activity were more in berries produced by tissue culture-derived plants compared to plants derived from runner cuttings (Debnath 2009). Faisal and Anis (2006) reported higher amount of chlorophyll *a* (0.91 ± 0.19 mg/g FW) and chlorophyll *b* (0.61 ± 0.09 mg/g FW) in micro propagated plants of *Psoralea corylifolia* compared to chlorophyll *a* (0.83 ± 0.31 mg/g FW) and chlorophyll *b* (0.53 ± 0.14 mg/g FW) in seedlings. Similarly Shahzad et al. (2007) found higher chlorophyll *a*/*b* ratio and carotenoid content in vitro regenerated plants compare to the seedlings of *Clitoria ternatea*. Saha Roy et al. (2010) found higher amount of β-carotene (0.07 μg/100 g) in banana fruit (*Musa paradisiaca*, AAB group) derived from tissue culture compare to vegetatively grown plants (0.03 μg/100 g). It is well known that cytokinin, BA, which is known to promote the shoot initiation in vitro*,* gave a carry-over effect promoting excessive vegetative growth of micro propagated field established plants (George and Sherrington 1984). Superior performance of the field grown tissue culture-derived plants was due to active shoot system of the plant which exists at the time of planting (Drew and Smith 1990). In our experiment, high chlorophyll, carotenoids and α-tocopherol content in micro propagated plants compare to conventionally grown plants was due to vigorous vegetative growth of micro propagated plants.

In conclusion, 4.44 μM BA was found to be optimal in producing maximum number of shoots per explants. Efficient in vitro rooting of individual shoot culture was obtained in 2.85 μM IAA plus 4.92 μM IBA treatment. Tissue culture-derived plants showed improved nutrient composition with respect to conventionally grown plants to contain higher amount lutein, β-carotene, α-tocopherol and total carotenoids.

## Abbreviations

BA: Benzyladenine
IAA: Indole-3-acetic acid
IBA: Indole-3-butyric acid
MS: Murashige and Skoog
NAA: α-naphthalene acetic acid
TRIA: Triacontanol

## References

[CR1] Darnoko D, Cheryan M, Moros E, Jerrel J, Perkins EG (2000). Simultaneous HPLC analysis of palm carotenoids and tocopherols using a C-30 column and photodiode array detector. J Liq Chrom Rel Technol.

[CR2] Debnath SC (2009). Characteristics of strawberry plants propagated by in vitro bioreactor culture and ex vitro propagation method. Eng Life Sci.

[CR3] Drew RA, Smith MK (1990). Field evaluation of tissue-cultured bananas in south-eastern Queensland. Aust J Exp Agr.

[CR4] Faisal M, Anis M (2006). Thidiazuron induced high frequency axillary shoot multiplication in *Psoralea corylifolia*. Biol Plantarum.

[CR5] Fuglie LJ (1999). The miracle tree: *Moringa oleifera:* natural nutrition for the tropics.

[CR6] George EF, Sherrington PD (1984). Plant propagation by tissue culture—handbook and directory of commercial laboratories.

[CR7] Islam S, Jahan MAA, Khatun R (2005). In vitro regeneration and multiplication of year-round fruit bearing *Moringa oleifera* L. J Biol Sci.

[CR8] Khan H, Siddique I, Anis M, Khan P (2011). In vitro organogenesis from internode derived callus cultures of *Capsicum annuum* L. J Plant Biochem Biotechnol.

[CR9] Lichtenthaler HK, Wellburn AR (1985). Determination of total carotenoids and chlorophylls *a* and *b* of leaf in different solvents. Biol Soc Trans.

[CR10] Marfori EC (2010). Clonal micropropagation of *Moringa oleifera* L. Philipp Agric Sci.

[CR11] Morton JF (1991). The horseradish tree, *Moringa pterygosperma* (Moringaceae)-a boon to arid lands. Econ Bot.

[CR12] Murashige T, Skoog FA (1962). Revised medium for rapid growth and bioassays with tobacco tissue cultures. Physiol Plantarum.

[CR13] Olson ME (2001). Combining data from DNA sequences and morphology for a phylogeny of Moringaceae (Brassicales). Syst Bot.

[CR14] Olson ME, Razafimandimbison SG (2000). *Moringa hildebrandtii* (Moringaceae): a tree extinct in the wild but preserved by indigenous horticultural practices in Madagascar. Adansonia.

[CR15] Palada MC (1996). Moringa *(Moringa oleifera* Lam.): a versatile tree crop with horticultural potential in the subtropical United States. Hort Sci.

[CR16] Saha Roy O, Bantawa P, Kumar Ghosh S, Teixeira da Silva JA, DebGhosh P, Mondal TK (2010). Micropropagation and field performance of *‘*Malbhog*’* (*Musa paradisiaca*, AAB group): a popular banana cultivar with high keeping quality of north east India. Tree For Sci Biotechnology.

[CR17] Shahzad A, Faisal M, Anis M (2007). Micro propagation through excised root culture of *Clitoria ternatea* and comparison between in vitro–regenerated plants and seedlings. Ann Appl Biol.

[CR18] Stephenson KK, Fahey JW (2004). Development of tissue culture methods for the rescue and propagation of endangered *Moringa* spp. germplasm. Econ Bot.

[CR19] Verdcourt B (1985). A synopsis of the Moringaceae. Kew Bull.

[CR20] Yapo E, Kouakou T, Kone M, Kouadio J, Kouame P, Merillon J-M (2011). Regeneration of pineapple (*Ananas comosus* L.) plant through somatic embryogenesis. J Plant Biochem Biotechnol.

